# HIF-1 activation induces doxorubicin resistance in MCF7 3-D spheroids via P-glycoprotein expression: a potential model of the chemo-resistance of invasive micropapillary carcinoma of the breast

**DOI:** 10.1186/1471-2407-12-4

**Published:** 2012-01-04

**Authors:** Sophie Doublier, Dimas C Belisario, Manuela Polimeni, Laura Annaratone, Chiara Riganti, Elena Allia, Dario Ghigo, Amalia Bosia, Anna Sapino

**Affiliations:** 1 Department of Genetics, Biology and Biochemistry, University of Turin, Via Santena, 5/bis, 10126 Turin, Italy; 2 Center of Experimental Research and Medical Sciences, University of Turin, Turin, Italy; 3 Department of Biomedical Sciences and Human Oncology, University of Turin, Turin, Italy

**Keywords:** HIF-1α, 3-D spheroids, Elastase, Invasive micropapillary breast carcinoma, Doxorubicin resistance, P-glycoprotein, MUC-1

## Abstract

**Background:**

Invasive micropapillary carcinoma (IMPC) of the breast is a distinct and aggressive variant of luminal type B breast cancer that does not respond to neoadjuvant chemotherapy. It is characterized by small pseudopapillary clusters of cancer cells with inverted cell polarity. To investigate whether hypoxia-inducible factor-1 (HIF-1) activation may be related to the drug resistance described in this tumor, we used MCF7 cancer cells cultured as 3-D spheroids, which morphologically simulate IMPC cell clusters.

**Methods:**

HIF-1 activation was measured by EMSA and ELISA in MCF7 3-D spheroids and MCF7 monolayers. Binding of HIF-1α to *MDR-1 *gene promoter and modulation of P-glycoprotein (Pgp) expression was evaluated by ChIP assay and FACS analysis, respectively. Intracellular doxorubicin retention was measured by spectrofluorimetric assay and drug cytotoxicity by annexin V-FITC measurement and caspase activity assay.

**Results:**

In MCF7 3-D spheroids HIF-1 was activated and recruited to participate to the transcriptional activity of *MDR-1 *gene, coding for Pgp. In addition, Pgp expression on the surface of cells obtained from 3-D spheroids was increased. MCF7 3-D spheroids accumulate less doxorubicin and are less sensitive to its cytotoxic effects than MCF7 cells cultured as monolayer. Finally, HIF-1α inhibition either by incubating cells with 3-(5'-hydroxymethyl-2'-furyl)-1-benzylindazole (a widely used HIF-1α inhibitor) or by transfecting cells with specific siRNA for HIF-1α significantly decreased the expression of Pgp on the surface of cells and increased the intracellular doxorubicin accumulation in MCF7 3-D spheroids.

**Conclusions:**

MCF7 breast cancer cells cultured as 3-D spheroids are resistant to doxorubicin and this resistance is associated with an increased Pgp expression in the plasma membrane via activation of HIF-1. The same mechanism may be suggested for IMPC drug resistance.

## Background

Invasive micropapillary carcinoma (IMPC) of the breast is a rare and aggressive histologic subtype of infiltrating breast carcinoma. It is characterized by small pseudopapillary clusters of cancer cells surrounded by clear spaces with loose fibrocollagenous stroma [1]. It has been reported that most IMPC are estrogen receptor (ER)-positive breast cancers displaying aberrant localization of the luminal glycoprotein mucin 1 (MUC1) at the stromal-basal surface of micropapillae, corresponding to an inversion of cell polarity [2]. IMPC frequently shows advanced stage at diagnosis, high incidences of lymphovascular invasion and axillary lymph node metastases, and high rates of local recurrence with short disease-free survival [3,4]. In addition, IMPC harbors genetic aberrations consistent with those of the luminal B subgroup of ER positive breast cancers [5], which are associated with poor outcome. Because of its highly aggressive behavior, preoperative neoadjuvant chemotherapy (NAC) has been considered for IMPC [6]. Interestingly, Alvarado-Cabrero et al. have recently reported that among a series of breast cancers treated with NAC none of the IMPC had a pathologic complete response to therapy, defined as the absence of any microscopic evidence of tumor in the mastectomy specimen and axillary lymph node dissection. Indeed, these patients had extensive residual invasive carcinoma after neoadjuvant therapy, and the disease was multifocal in most of the cases [7]. To our knowledge no data have been reported on the molecular basis of this phenomenon that may be related, at least in part, to multidrug resistance (MDR) of the tumor emboli.

The development of MDR is a major obstacle to cancer treatment. MDR in cancer cells produces resistance to the cytotoxic effects of numerous antineoplastic drugs that are structurally and mechanistically unrelated, and this significantly decreases the efficacy of cancer chemotherapy [8]. MDR may be either constitutive or acquired and can be mediated by several different mechanisms including target alterations, enhanced DNA repair, evasion of apoptosis, induction of drug-metabolizing enzymes, alterations in drug uptake and active transport of drugs out of cells [9]. Energy-dependent efflux of chemotherapeutic drugs out of cells is to a great extent mediated by transmembrane transporters belonging to the ATP-binding cassette (ABC) proteins superfamily, which use ATP hydrolysis as an energy source to transport a variety of structurally diverse molecules across membranes irrespective of concentration gradient [10]. Among the 48 members of the human ABC transporters family, P-glycoprotein (Pgp, ABCB1) is the most widely studied. A great deal of studies has coupled increased expression of Pgp to impairment of chemotherapy response and lower patient survival [11].

There is a general consensus that hypoxia in the centre of solid tumors dramatically decreases the chemosensitivity of tumor cells and that experimental hypoxia promotes drug resistance to anticancer agents in a variety of cell lines [12]. A central component of hypoxic adaptation is hypoxia-inducible factor-1 (HIF-1), the transcription factor that leads hypoxic cells to up-regulate proteins that promote survival and increases aggressiveness of hypoxic tumor cells [13,14]. When adequate oxygen (O_2_) is present, the subunit HIF-1α becomes hydroxylated at several proline residues and this leads to ubiquitination and proteasomal degradation. However, when O_2 _is absent this molecule survives and translocates to the nucleus, where it forms an heterodimer with HIF-1β. The HIF-1 heterodimer is a transcription factor recognized by at least 100 hypoxia response element (HRE) sites on chromosomes [15]. In particular, it has been recently demonstrated that the activation of HIF-1 is associated with an increase of Pgp expression and the occurrence of MDR in tumor cells [16-20].

In this study, to investigate the role of drug resistance in the aggressive behavior of tumor cell clusters encountered in IMPC, we used a model of three-dimensional cell culture, defined as 3-D spheroids, which has been described previously [21]. Here we show that the ER-positive MCF7 breast cancer cells cultured as 3-D spheroids are resistant to doxorubicin and that this resistance is associated with an increased Pgp expression at the plasma membrane via activation of HIF-1.

## Methods

### Cells

MCF7 human breast cancer epithelial cells were cultured in a 1:1 mixture of DMEM/HAM'S F12 medium (Gibco, Paisley, UK) supplemented with 10% (v/v) fetal bovine serum (FBS) (Sigma-Aldrich S.r.l., Milan, Italy), 1% penicillin/streptomycin (Sigma), 1% L-glutamine (Sigma), and maintained in a humidified incubator at 37°C, 5% CO_2 _and 20% O_2_. For the different experimental conditions, aliquots of MCF7 monolayer cells were either cultured in a humidified incubator at 37°C, 5% CO_2_, and 20% O_2 _(basal condition), or 3% O_2 _for 24 h (hypoxic exposure). Hypoxic exposure of MCF7 cells was used as a positive control of HIF-1 activation since we did not measure the gradient in O_2 _in MCF7 3-D spheroids. Preliminary experiments were performed to set the pressure of O_2 _necessary to detect HIF-1α expression by immunoblot assay and HIF-1 DNA-binding activity by EMSA and DNA binding ELISA (data not shown). We have determined that a decrease of O_2 _concentration to 3% was sufficient to induce HIF-1 activation in MCF7 cells. Other aliquots of MCF7 monolayers cells were transfected with *MDR-1 *gene, previously cloned in pCDNA3, using jetPEI transfection reagent (Poly-plus Transfection, Euroclone, Pero, Italy) according to the manufacturer's instructions in order to obtain an overexpression of Pgp, or incubated with human neutrophil elastase (Calbiochem, San Diego, CA, 21 U/mg) in order to obtain MCF7 3-D spheroids, as described previously [21]. Briefly, 5 × 10^6 ^MCF7 cells were plated in 100-mm diameter Petri dishes and supplemented with DMEM/HAM'S F12 medium containing 1% FBS and 20 μg/ml human neutrophil elastase. Compact multicellular MCF7 3-D spheroids with smooth margins were observed after a 24-48 h-incubation. Spheroids of various sizes were obtained and pooled to perform the different experiments.

### Western blot analysis

Nuclear extracts (60 μg), obtained with the Nuclear Extraction Kit (Active Motif, Vinci-Biochem, Florence, Italy), were separated by SDS-PAGE, transferred to PVDF membrane (Immobilon-P, Millipore, Bedford, MA). PVDF membrane was blocked in Tris-buffered saline solution (TBS)-nonfat dry milk 5% overday at 4°C and probed with anti-HIF-1α mouse mAb (diluted 1:500 in TBS-nonfat dry milk 5%, cat # 610959, BD Biosciences, San Jose, CA) and anti-PCNA mouse mAb (diluted 1:500 in TBS-nonfat dry milk 5%, sc-56, Santa Cruz Biotechnology, Santa Cruz, CA) overnight at 4°C.

### Electrophoretic mobility shift assay (EMSA)

Nuclear extracts (10 μg) were used to detect HIF-1α translocation. The probe containing the HIF-1α oligonucleotide consensus sequence was labeled with [γ-^32^P]ATP (Perkin Elmer) (3000 Ci/mmol, 250 μCi), using T4 polynucleotide kinase (Roche, Basel, Switzerland). The sequence of oligonucleotide was: 5'-TCTGTACGTGACCACACTCACCTC-3'; 3'-AGACATGCACTGGTGTGAGTGGAG-5'. The DNA-protein complex was separated on a non-denaturating 4% polyacrylamide gel in TBE buffer (0.4 Mol/L Tris, 0.45 Mol/L boric acid, 0.5 Mol/L EDTA, pH 8.0). After electrophoresis, the gel was dried and autoradiographed by exposure to X-ray film.

### DNA binding enzyme-linked immunosorbent assay (ELISA)

Nuclear extracts (10 μg), obtained with the Nuclear Extraction Kit (Active Motif, Vinci-Biochem), were used to detect HIF-1 capacity to bind an HRE by TransAM ELISA Kit (Active Motif, Vinci-Biochem) according to the manufacturer's instructions. Briefly, samples were added to a 96-well plate, on which there was an immobilized oligonucleotide containing an HRE (5'-TACGTGCT-3') from the *EPO *gene, incubated with anti-HIF-1α antibody (diluted 1:1000 in Antibody Binding Buffer), subjected to a horseradish peroxidase-conjugated anti-mouse antibody (diluted 1:1000 in Antibody Binding Buffer) and then incubated with Developing Solution, until the blue color development reaction was performed. Absorbance was read on a Packard EL340 microplate reader (Bio-Tek Instruments, Winooski, VT) at 450 nm with a reference wavelength of 655 nm; the absorbance values were expressed as mU optical density/μg nuclear proteins.

### Semi-quantitative polymerase chain reaction

Total RNA was obtained by the guanidinium thiocyanate-phenol-chloroform method [22]. Two μg of total RNA were reversely transcribed into cDNA, in a final volume of 20 μl, using the QuantiTect Reverse Transcription Kit (Qiagen, Germantown, USA) according to the manufacturer's instructions. Semi-quantitative PCR was carried out in a final volume of 50 μl with specific primers for the quantitation of *pyruvate dehydrogenase kinase isozyme 1 *(*PDK1*, p1 primer: 5'-GAAGCAGTTCCTGGACTTCG-3'; p2 primer: 5'-ACCAATTGAACGGATGGTGT-3'; 56°C, 153 bp), *phosphoglycerate kinase 1 *(*PGK1*, p1 primer: 5'-TCTCATGGATGAGGTGGTGA-3'; p2 primer: 5'-CTTCCAGGAGCTCCAAACTG-3'; 56°C, 152 bp), *vascular endothelial growth factor *(*VEGF*, p1 primer: 5'-ATCTTCAAGCCATCCTGTGTGC-3'; p2 primer: 5'-GCTCACCGCCTCGGCTTGT-3'; 62°C, 488 bp) and *ribosomal protein S14 *genes (p1 primer: 5'-AGGTGCAAGGAGCTGGGTAT-3'; p2 primer: 5'-TCCAGGGGTCTTGGTCCTATTT-3'; 60°C, 77 bp). PCR amplification was 1 cycle of denaturation at 95°C for 3 min, 30 cycles of denaturation at 94°C for 30 s, annealing for 30 s, synthesis at 72°C for 1 min. Differences in expression levels between the different experimental conditions (MCF7 control cells, MCF7 hypoxic cells and MCF7 3-D spheroids in the presence or absence of YC-1) were large enough to be detected by semi-quantitative PCR. To determine the assay range, we plotted increasing numbers of PCR cycles against the integrated optical density obtained from computer-aided densitometry (Image J 1.43u Java 1.6.0_10, National Institutes of Health, Bethesda, MD, USA, http://imagej.nih.gov/ij) (data not shown), as described previously [23]. The PCR products were separated on a 4% agarose gel and visualized with ethidium bromide.

### Chromatin immunoprecipitation assay (ChIP)

The chromatin immunoprecipitation assay was done following the recommendations of the manufacturer (Upstate, Billerica, MA) with some modifications. Briefly, cells were plated in 100-mm diameter dishes (6 × 10^6 ^cells per dish); after 24 h, they were incubated with formaldehyde (final concentration, 1%) for 10 min at 37°C to cross-link proteins to DNA. The cross-linking reaction was quenched by the addition of one-tenth volume of 1.25 mol/L glycine, giving 125 mmol/L final concentration. Cells were washed twice with ice-cold PBS 1X, resuspended in radioimmunoprecipitation assay buffer [150 mMol/L NaCl, 1% NP40, 0.5% deoxycholate, 0.1% SDS, 5 mMol/L EDTA, 50 mMol/L Tris-HCl (pH 8.0)] containing 1 mMol/L PMSF, 1 μg/mL aprotinin, and 1 μg/mL pepstatin A, and kept on ice for 30 min. Then, cell lysates were sonicated on ice with a Hielscher UP200S ultrasound sonicator (3 × 40 s, amplitude 40%; Hielscher Ultrasonics GmbH, Germany) until cross-linked chromatins were sheared to yield DNA fragments between 200 and 1,000 bp. One tenth of whole lysate was used to quantify the amount of DNA present in different samples and considered as "input DNA". Supernatants were incubated with salmon sperm DNA/protein agarose-50% slurry to reduce non-specific background. Immunoprecipitation was then done overnight at 4°C with 5 μg of anti-HIF-1α antibody [rabbit polyclonal, (H-206): sc-10790, Santa Cruz Biotechnology] or without antibody (negative control). These supernatants were supplemented with 5 Mol/L NaCl and heated overnight at 65°C to reverse protein-DNA cross-links. The immunocomplexes were further treated with DNase-and RNase-free proteinase K, and DNA was purified by phenol/chloroform extraction and ethanol precipitation. Semi-quantitative PCR was performed with specific primers flanking the putative HRE within the promoter region of the human *MDR-1 *gene (named "Pgp+", p1 primer: 5'-GGAGCAGTCATCTGTGGTGA-3'; p2 primer: 5'-CTCGAATGAGCTCAGGCTTC-3'), primers flanking a *MDR-1 *promoter region that does not contain HRE (named "Pgp-", used as a negative control, p1 primer: 5'-GAAGGTCTTCCCAGTAACCTACC-3'; p2 primer: 5'-GCCAGAGTTGAGAAGTTTAGCC-3'), primers flanking the HRE of the human vascular endothelial growth factor (VEGF) promoter (used as a positive control, p1 primer: 5'-GCCTCTGTCTGCCCAGCTGC-3'; p2 primer: 5'-GTGGAGCTGAGAACGGGAAGC-3'), as reported previously [24]. Differences in expression levels between the different experimental conditions (MCF7 control cells, MCF7 hypoxic cells and MCF7 3-D spheroids in the presence or absence of YC-1) were large enough to be detected by semi-quantitative PCR. To determine the assay range, we plotted increasing numbers of PCR cycles against the integrated optical density obtained from computer-aided densitometry (Image J 1.43u Java 1.6.0_10, National Institutes of Health, Bethesda, MD, USA, http://imagej.nih.gov/ij) (data not shown), as described previously [23]. Cycling for "Pgp+" and "Pgp-" was: 1 cycle at 95°C for 7 min, followed by 37 cycles at 94°C for 1 min, annealing at 56°C for 1 min, extension at 72°C for 1 min; cycling for VEGF was: 1 cycle at 95°C for 7 min, followed by 37 cycles at 94°C for 1 min, annealing at 64°C for 1 min, extension at 72°C for 1 min. The PCR products were separated on a 4% agarose gel and visualized with ethidium bromide.

### HIF-1α siRNA transfection

Cells were transfected with specific HIF-1α siRNA in order to obtain a knockdown (KD) phenotype. Cells were plated in a six well tissue culture plate (200,000 cells/well) and cultured in antibiotic-free DMEM/HAM'S F12 medium containing 10% FBS. After 24 h cells were washed once with siRNA Transfection Medium (Santa Cruz Biotechnology) and incubated for 6 h with 1 ml siRNA transfection medium, containing 6 μl of siRNA Transfection Reagent (sc-29528, Santa Cruz Biotechnology), and 400 nM/L of HIF-1α siRNA (sc-35561, Santa Cruz Biotechnology). In each set of experiments one dish was treated with 400 nM/L of Control siRNA-A (Santa Cruz Biotechnology), a non-targeting 20-25 nucleotides siRNA designed as a negative control, instead of HIF-1α siRNA. At the end of the incubation, 1 ml of DMEM/HAM'S F12 medium containing 1% penicillin/streptomycin and 20% FBS was added for 24 h. Subsequently, MCF7 cell monolayers under normoxic conditions (MCF7 control cells) and MCF7 cell monolayers under hypoxic conditions were washed and cultured for 48 h in DMEM/HAM'S F12 medium with 1% penicillin/streptomycin and 10% FBS. To form spheroids, 4 wells of a six well tissue culture plate were trypsinized and plated in 60-mm diameter Petri dishes and supplemented with DMEM/HAM'S F12 medium containing 1% FBS and 20 μg/ml human neutrophil elastase. Compact multicellular MCF7 3-D spheroids with smooth margins were observed after a 24-48 h-incubation. To verify the siRNA efficacy, cells were lysed and the expression of HIF-1α protein was analysed by Western blotting using an anti-HIF-1α mouse monoclonal antibody (mAb) (diluted 1:500 in TBS-nonfat dry milk 5%, cat # 610959, BD Biosciences). To assess the siRNA specificity for HIF-1α, we checked the expression of PCNA, a product of an housekeeping gene in both transfected and untransfected cells (data not shown). Silenced cells were indicated as HIF-1α KD 3-D spheroids, HIF-1α KD Monolayer Hypoxia, HIF-1α KD Monolayer Normoxia Pgp+, and HIF-1α KD Monolayer Normoxia.

### Pgp expression

Pgp proteins were labeled on the surface of non-permeabilized cells and quantified by FACS analysis. Cells were fixed with cold 2% paraformaldehyde solution for 15 min, washed in PBS 1X and then resuspended in PBS 1X supplemented with 0.25% BSA/0.01% sodium azide containing an anti-Pgp polyclonal antibody [1:20 diluted, Mdr (H-241): sc-8313, Santa Cruz Biotechnology] or an isotype-matched negative control (DakoCytomation, Glostrup, Denmark) for 45 min at 4°C. Cells were then washed 3 times with PBS 1X/0.25% BSA/0.01% sodium azide and incubated with a FITC-conjugated anti-rabbit antibody (1:400 diluted) for 30 min at 4°C. Samples were then collected and analyzed using a FACSCalibur CellQuest (Becton Dickinson). Cells were electronically gated according to their light-scattering properties to exclude cell debris. Ten thousand cells were analyzed at each experimental point. The percentage of cells positive for Pgp was calculated by the Cell Quest software (Becton Dickinson).

### Doxorubicin accumulation

Cellular doxorubicin content was evaluated by fluorimetric assay. Before every test, cells were washed and then incubated in fresh medium containing 3 μMol/L doxorubicin (Sigma) for 18 h. Intracellular doxorubicin content was measured using a Perkin-Elmer LS-5 fluorimeter (Perkin Elmer, Shelton, CT) at 475 nm (excitation) and 553 nm (emission); fluorescence was converted in ng of doxorubicin per mg of cellular protein, using a calibration curve prepared previously. The protein content of cell monolayers was assessed with the bicinchoninic acid (BCA) protein assay kit (Sigma). To evaluate the contribution of Pgp in the drug resistance of MCF7 3-D spheroids, we measured the drug accumulation in the presence 100 μMol/L Pgp inhibitor verapamil hydrochloride (Sigma).

### Detection of apoptosis

Induction of apoptosis due to the cytotoxic effect of doxorubicin was evaluated by Annexin V-fluorescein isothiocyanate (FITC) apoptosis detection kit (Sigma) and by measuring the caspase activity:

### Annexin V-FITC measurement

Cells were incubated in the absence or presence of doxorubicin (3 μMol/L for 18 h), washed with phosphate-buffered saline solution (PBS) 1X, detached with trypsin/EDTA, and resuspended in 1 ml of binding buffer (100 mMol/L HEPES/NaOH, 140 mMol/L NaCl, 25 mMol/L CaCl_2_, pH 7.5). These cell suspensions were then incubated for 10 min with 5 μl annexin V-FITC conjugate (0.05 mg/ml). Cells were washed 3 times with fresh PBS 1X and fluorescence of each sample was evaluated. The fluorescence level of annexin V was recorded using a FACSCalibur system (Becton Dickinson, Bedford, MA) reading the emission selected by a 530 nm band pass filter; the percentage of cells positive for annexin V was calculated by the Cell Quest software (Becton Dickinson). Results are expressed as average of percent of control values obtained from triplicate experiments.

### Caspase activity assays

The caspase activity was evaluated by fluorogenic assay (Sigma). Cells and spheroids were incubated with doxorubicin (3 μMol/L for 18 h) in the absence or in the presence of YC-1 (5 μMol/L) washed with PBS 1X, lysed in 1 ml of lysis buffer (20 mMol/L HEPES/KOH, 10 mMol/L KCl, 1.5 mMol/L MgCl_2_, 1 mMol/L EGTA, 1 mMol/L EDTA, 1 mM/L DTT, 1 mM/L PMSF, 10 μg/ml leupeptin, pH 7.5), detached with a scraper and centrifuged at 13000 r.p.m for 15 min at 4°C, the supernatant was collected and the protein content was assessed with the BCA protein assay kit (Sigma). Briefly, cell lysates (10 μg) were incubated with 20 μMol/L of substrate in a caspase substrate buffer (25 mM/L HEPES, 0.1% w/v CHAPS, 10% w/v sucrose, 10 mM/L DTT, 0.1% w/v egg albumin, pH 7.5) for 1 h at 37°C, then the reaction was stopped with 0.1% w/v ice-cold TCA. The content of substrate cleavage was measured using a Perkin-Elmer LS-5 fluorimeter (Perkin Elmer) at 380 nm (excitation) and 460 nm (emission); fluorescence was converted in μmol AMC (7-amino-4-methyl coumarin) per mg of cellular protein, using a calibration curve prepared previously.

### Immunohistochemistry analysis

On formalin fixed paraffin embedded sections of cells and tissues, we performed morphological and immunohistochemical analysis to evaluate the similarities between MCF7-3D spheroids and IMPC. Cells were fixed in 10% buffered formalin for 5 min, transferred in a tube and centrifuged for 5 min at 800 rpm. Cell pellets were rinsed 2 times in PBS and re-fixed in 10% formalin for 15 min. Cell blocks were prepared following the standard processing (vacuum processor, Leica ASP 300S, Leica AG, Switzerland) and paraffin embedded. For all specimens, 3 μm thick sections were cut. Both MCF7 cell pellets and breast biopsies from patients with IMPC of the breast (used as a positive control of MUC1, CD44 and p53 expression) were processed for immunohistochemical staining. Sections were deparaffinized and submitted to endogenous peroxidase activity blocking with 3% hydrogen peroxide for 7 min. An antigen retrieval procedure was performed using citrate buffer solution, pH 6.0, for 40 min. Sections were then incubated with an anti-MUC1 monoclonal antibody (mAb) (clone Ma695) (Novocastra, Newcastle Upon Tyne, UK) diluted 1:100, for 30 min. Cells were then washed 3 times with PBS 1X and incubated with anti-mouse labeled polymer HRP-conjugated (Envision + System, Dako Glostrup, Denmark) for 30 min. Then 3,3-diaminobenzidine (DAB, Dako) was used as a chromogen to reveal antibody binding and slides were counterstained in Mayer's haematoxylin (BioGenex Laboratories) for 30 seconds, dehydrated and mounted. Tissue sections and MCF7 3D-spheroids were immunostained for HIF-1α using the anti-HIF-1α polyclonal antibody (ab2185, Abcam, Cambridge, UK) with the automated stainer BenchMark XT (Ventana-Roche F, Hoffmann-La Roche Ltd, Tucson, AZ). In addition, to evaluate the expression of biological markers of aggressiveness, MCF7 control cells and MCF7 3D-spheroids were stained using the BenchMark XT by anti-CD44 (DF1485, DAKO), a pleiotropic molecule involved in cell adhesion and tumor metastasis formation, and anti-p53 antibodies (DO-7 prediluted Ventana), since CD44 and p53 are two molecules frequently expressed in IMPC [25,26]. All incubations were performed at room temperature. Control experiments included incubation of sections or cells with non-immune isotypic control antibodies or the omission of primary antibodies followed by the appropriate labeled secondary antibodies.

### Statistical analysis

Measurements were performed in triplicate. All data in text and figures are provided as means ± SEM. The results were analyzed by a one-way Analysis of Variance (ANOVA) and Tukey's test. p < 0.05 was considered significant.

## Results

### HIF-1 is activated in MCF7 3-D spheroids

**Figure 1:**
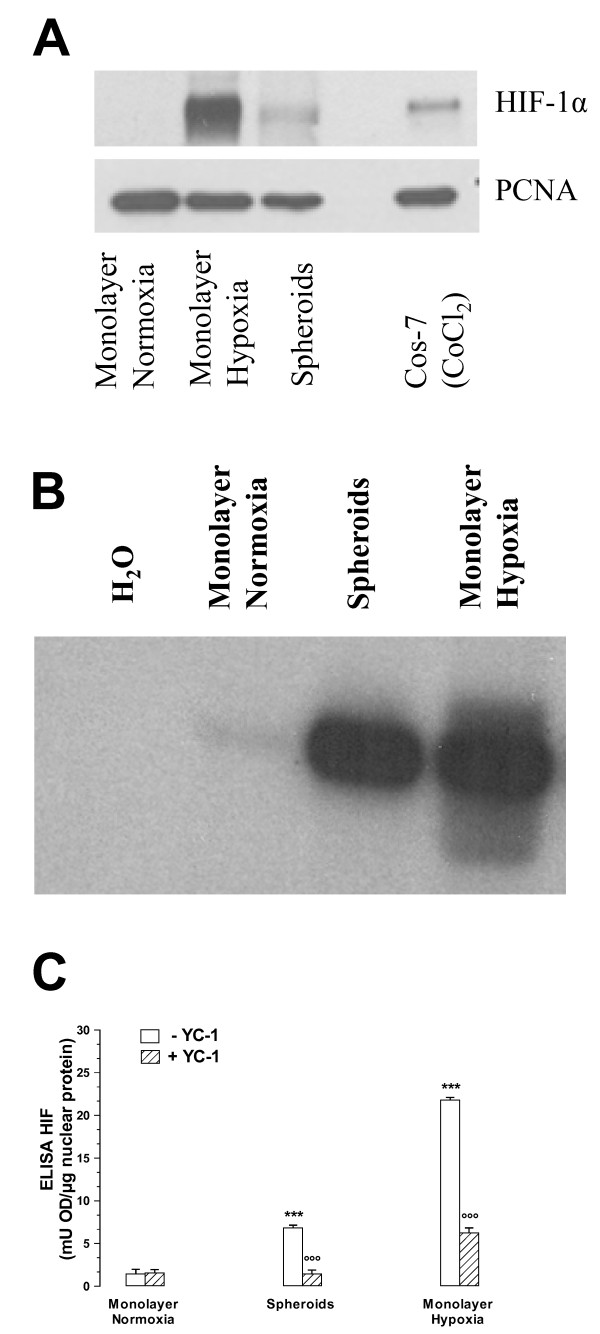
**Effects of MCF7 3-D spheroid formation on HIF-1 activation**. **A: **Representative gel of Western blot analysis of HIF-1α performed on nuclear cellular extracts (see Materials and Methods for details) in MCF7 cell monolayers under normoxic conditions (Monolayer Normoxia), MCF7 cell monolayers under hypoxic conditions (Monolayer Hypoxia), MCF7 3-D spheroids (Spheroids), and Cos-7 cells stimulated with CoCl_2 _(used as a positive control of HIF-1α expression). **B: **Representative gel of EMSA for detection of HIF-1α nuclear translocation. Monolayer Normoxia: MCF7 cell monolayers under normoxic conditions; Spheroids: MCF7 3-D spheroids; and Monolayer Hypoxia: MCF7 cell monolayers under hypoxic conditions as positive control (3% O_2 _for 24 h). In each experiment one lane was loaded with bisdistilled water only (H_2_O) in place of cellular extracts as negative control. This figure is representative of three similar experiments. **C: **Detection of HIF-1 activation by DNA binding ELISA in MCF7 cell monolayers under normoxic conditions (Monolayer Normoxia); MCF7 3-D spheroids (Spheroids); and MCF7 cell monolayers under hypoxic conditions (Monolayer Hypoxia). Cell monolayers and spheroids were incubated in the absence (white columns) or in the presence (hatched columns) of 5 μM YC-1 for 24 h. ***, P < 0.0001 versus Monolayer Normoxia without YC-1; °°°, P < 0.0001 versus Spheroids or Monolayer Hypoxia without YC-1. This figure is representative of three similar experiments, performed in duplicate.

**Figure 2:**
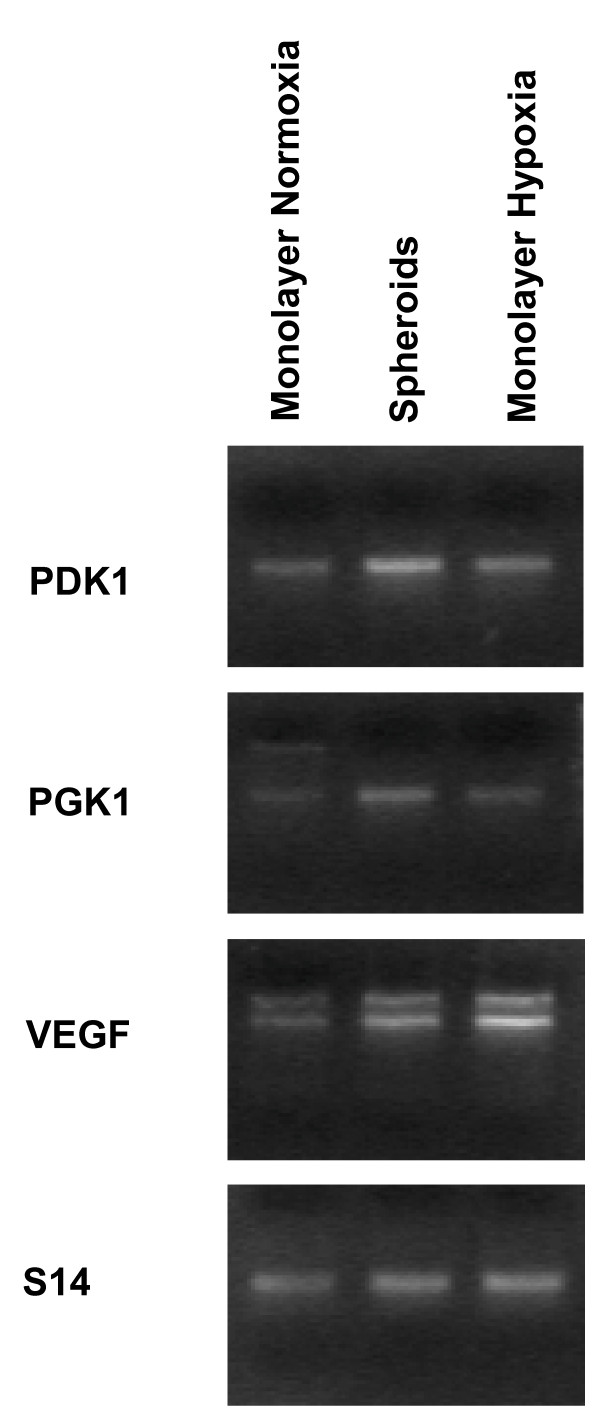
**Relative expression of *PDK1, PGK1, VEGF *(well-known HIF-1 target genes) and *S14 *(housekeeping gene) checked by semi-quantitative PCR analysis in MCF7 cell monolayers under normoxic conditions (Monolayer Normoxia); MCF7 3-D spheroids (Spheroids); and MCF7 cell monolayers under hypoxic conditions (Monolayer Hypoxia)**. The figure is representative of three similar experiments.

Cells cultured as 3-D spheroids are associated with a microenvironment characterized by gradients in O_2_, pH, and nutrients [27,28]. Therefore, we investigated whether in vitro MCF7 3-D spheroid formation may lead to the activation of HIF-1, a key component of hypoxic adaptation. We first showed with western blot analysis that HIF-1α was present into the nucleus of cells from MCF7 3-D spheroids and MCF7 cell monolayers under hypoxic conditions (MCF7 hypoxic cells, used as a positive control of HIF-1 activation), whereas HIF-1α was undetectable in MCF7 cell monolayers under normoxic conditions (MCF7 control cells) (Figure 1a). We then observed with EMSA that HIF-1α was translocated into the nucleus of cells from MCF7 3-D spheroids and MCF7 hypoxic cells, whereas MCF7 control cells showed undetectable levels of HIF-1α (Figure 1b). These results were confirmed by DNA binding ELISA: MCF7 control cells exhibited a minimal activation of HIF-1, while MCF7 3-D spheroids and MCF7 hypoxic cells showed a significant increase in the transcription factor activation, defined by an increased capacity to bind HRE (Figure 1c). The incubation of cells with a widely used HIF-1α inhibitor, 3-(5'-hydroxymethyl-2'-furyl)-1-benzylindazole (YC-1) (5 μMol/L for 24 h), which induces a decrease in HIF-1α accumulation by down-regulating HIF-1α mRNA translation [29], significantly decreased HIF-1 activation in both MCF7 3-D spheroids and MCF7 hypoxic cells, while, as expected, it had no effect in MCF7 control cells (Figure 1c). We finally observed with RT-PCR analysis that the expression of the genes *PDK1, PGK1*, and *VEGF*, which are well-known targets of HIF-1, was increased in MCF7 3-D spheroids and MCF7 hypoxic cells compared to MCF7 control cells (Figure 2).

### Binding of HIF-1α to *MDR-1 *gene promoter and increased expression of Pgp in MCF7 3-D spheroids and in MCF7 hypoxic cells

**Figure 3:**
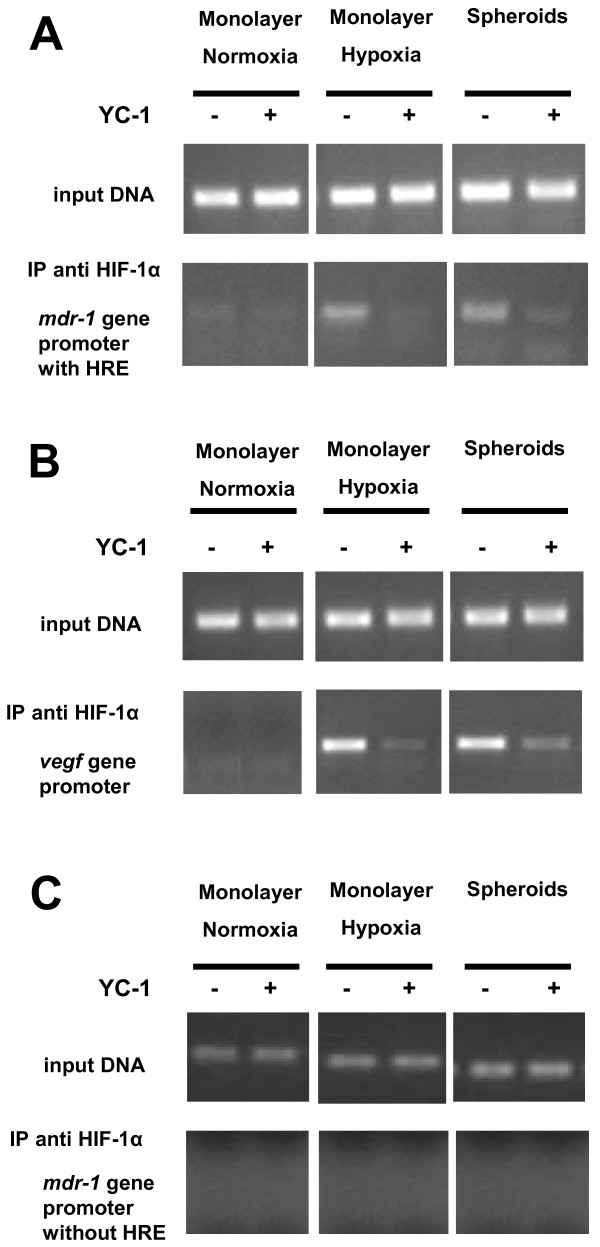
**HIF-1α binds to HRE within *MDR-1 *and *VEGF *gene promoters in MCF7 3-D spheroids**. The binding of HIF-1α on *MDR-1 *and *VEGF *gene promoters were measured by ChIP assay as described in Materials and Methods. **A: **PCR analysis for the *MDR-1 *gene promoter region containing a HRE was performed on immunoprecipitation samples (IP) with anti-HIF-1α antibody and with purified total input DNA (input DNA) from MCF7 control cells (Monolayer Normoxia); MCF7 hypoxic cells (Monolayer Hypoxia); and MCF7 3-D spheroids (Spheroids). **B: **PCR analysis for the *VEGF *gene promoter (an established HIF-1 target gene) was performed on immunoprecipitation samples with anti-HIF-1α antibody and with purified total input DNA from MCF7 control cells; MCF7 hypoxic cells; and MCF7 3-D spheroids. **C**. PCR analysis for the *MDR-1 *gene promoter region that does not contain a HRE was performed on immunoprecipitation samples (IP) with anti-HIF-1α antibody and with purified total input DNA from MCF7 control cells; MCF7 hypoxic cells; and MCF7 3-D spheroids. Cell monolayers and spheroids were incubated in the absence (*-*) or in the presence (*+*) of 5 μM YC-1 for 24 h. The figures are representative of three similar experiments.

We then evaluated in MCF7 3-D spheroids and in MCF7 hypoxic cells whether HIF-1α may bind to a putative HRE within the promoter region of the *MDR-1 *gene, increasing Pgp transcription, by a ChIP assay. After precipitation of cell lysates with a specific anti-HIF-1α antibody, the *MDR-1 *gene promoter containing a HRE and *VEGF *gene promoter (an established HIF-1 target gene) were amplified by PCR with specific primers. In MCF7 3-D spheroids and in MCF7 hypoxic cells, occupancy of HIF-1α at the *MDR-1 *(Figure 3a) and *VEGF *(Figure 3b) gene promoters was higher than in MCF7 control cells. We checked in all conditions tested that chromatin precipitated with an anti-HIF-1α antibody showed no enrichment of the *MDR-1 *promoter region that does not contain a HRE (Figure 3c). Moreover, incubation of cells with YC-1 (5 μMol/L for 24 h) was able to reduce the binding of HIF-1α to *MDR-1 *and *VEGF *gene promoters in MCF7 3-D spheroids and MCF7 hypoxic cells (Figure 3a and 3b). These results suggest that, after activation, HIF-1 is recruited to participate to the transcriptional activity of *MDR-1 *gene in both MCF7 3-D spheroids and MCF7 hypoxic cells.

**Figure 4:**
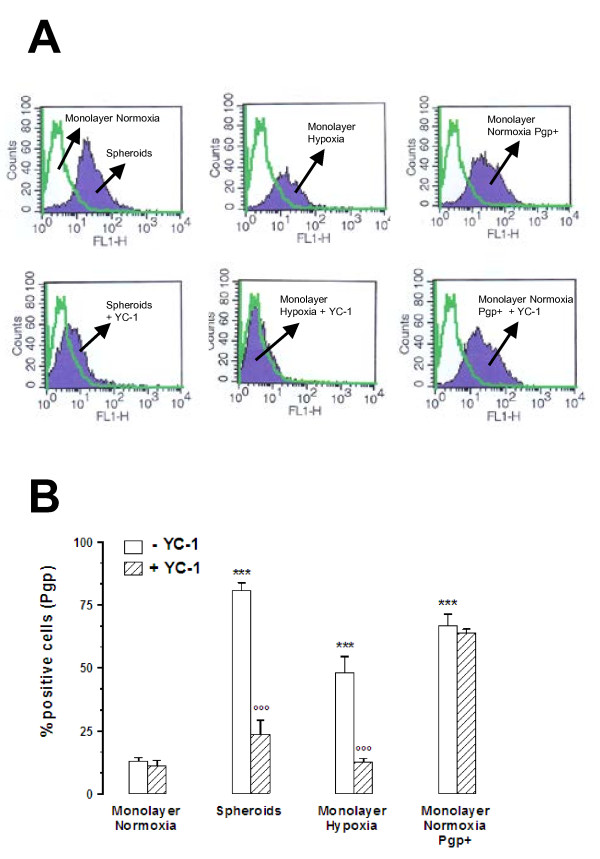
**Effects of MCF7 3-D spheroid formation and hypoxia on Pgp expression**. Pgp protein levels were labeled on the surface of non-permeabilized cells and were quantified by FACS analysis as reported in Materials and Methods. **A: **Pgp protein levels were higher in MCF7 3-D spheroids (Spheroids), in MCF7 hypoxic cells (Monolayer Hypoxia), and in MCF7 cells transfected with MDR-1 gene (Monolayer Normoxia Pgp+) compared to Pgp protein levels in MCF7 control cells (Monolayer Normoxia). The incubation of cells with YC-1 (5 μMol/L for 24 h) decreased the Pgp surface expression in both MCF7 3-D spheroids and MCF7 hypoxic cells, while it had no effect in Pgp + cells. These figure panels are representative of three similar experiments. B: The percentage of positive cells for Pgp in MCF7 cells cultured as monolayers under normoxic conditions (Monolayer Normoxia), 3-D spheroids (Spheroids), monolayers under hypoxic conditions (Monolayer Hypoxia), and transfected with MDR-1 gene (Monolayer Normoxia Pgp+). Cells and spheroids were incubated in the absence (white columns) or in the presence (hatched columns) of 5 μM YC-1 for 24 h. ***, P < 0.0001 versus Monolayer Normoxia without YC-1; °°°, P < 0.0001 versus Spheroids or Monolayer Hypoxia without YC-1.

**Figure 5:**
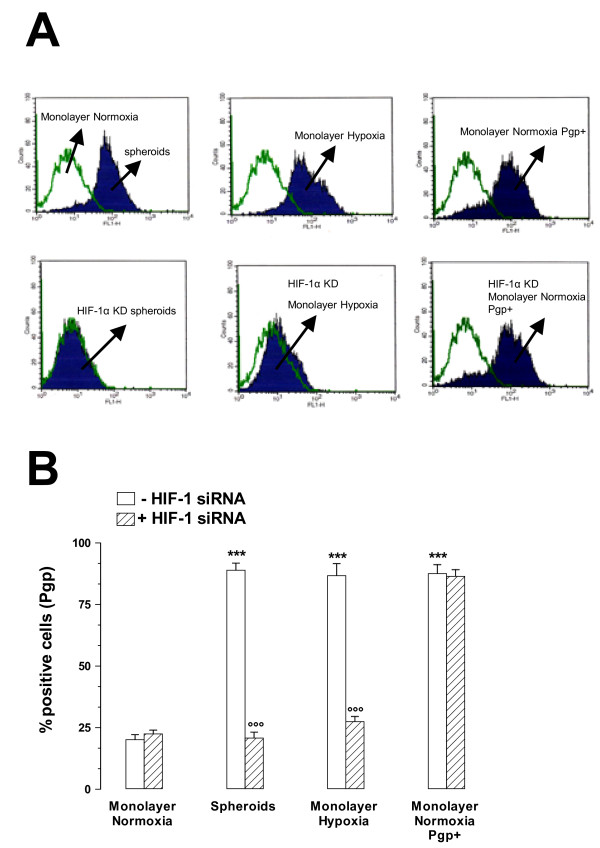
**Effects of HIF-1α silencing on Pgp expression in MCF7 3-D spheroids and MCF7 hypoxic cells**. Pgp protein levels were labeled on the surface of non-permeabilized cells and were quantified by FACS analysis as reported in Materials and Methods. **A: **Pgp protein levels were higher in MCF7 3-D spheroids (Spheroids), in MCF7 hypoxic cells (Monolayer Hypoxia), and in MCF7 cells transfected with *MDR-1 *gene (Monolayer Normoxia Pgp+) compared to Pgp protein levels in MCF7 control cells (Monolayer Normoxia). The transfection of cells with specific siRNA for HIF-1α (HIF-1α knockdown, KO) significantly decreased the Pgp surface expression in both MCF7 3-D spheroids and MCF7 hypoxic cells, while it had no effect in Pgp + cells. Silenced cells were indicated as HIF-1α KD 3-D Spheroids, HIF-1α KD Monolayer Hypoxia and HIF-1α KD Monolayer Normoxia Pgp+. These figure panels are representative of three similar experiments. B: The percentage of positive cells for Pgp in MCF7 cells cultured as monolayers under normoxic conditions (Monolayer Normoxia), 3-D spheroids (Spheroids), monolayers under hypoxic conditions (Monolayer Hypoxia), and transfected with MDR-1 gene (Monolayer Normoxia Pgp+). Cells and spheroids were transfected with 400 nM/L of HIF-1α (hatched columns) or transfected with vehicle alone (white columns). ***, P < 0.0001 versus Monolayer Normoxia transfected with vehicle alone; °°°, P < 0.0001 versus Spheroids or Monolayer Hypoxia transfected with vehicle alone.

Pgp protein levels were then quantified by FACS analysis on the surface of non-permeabilized cells. The percentage of positive cells for Pgp was significantly higher in MCF7 3-D spheroids, in MCF7 hypoxic cells, and in MCF7 cells transfected with MDR-1 gene (MCF7 Pgp + cells, used as a positive control of Pgp expression) compared to the percentage of positive cells for Pgp in MCF7 control cells (Figure 4a and 4b). The inhibition of HIF-1α either by incubating cells with YC-1 (5 μMol/L for 24 h) (Figure 4a and 4b) or by transfecting cells with specific siRNA for HIF-1α (Figure 5a and 5b) significantly decreased the Pgp surface expression in both MCF7 3-D spheroids and MCF7 hypoxic cells, while it had no effect in MCF7 control cells or in MCF7 Pgp + cells. These results suggest that in both MCF7 3-D spheroids and MCF7 hypoxic cells, surface Pgp expression is associated with HIF-1 activation.

### MCF7 3-D spheroids are more resistant to doxorubicin than MCF7 cell monolayers under normoxic conditions: they accumulate less doxorubicin and are less sensitive to its cytotoxic effects

**Figure 6:**
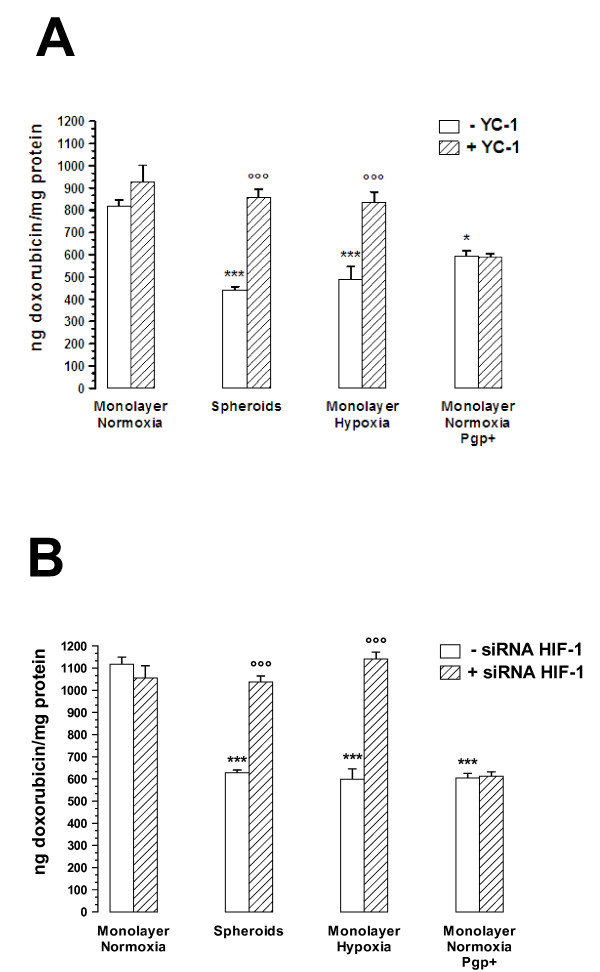
**Effects of 3-D spheroid formation and hypoxia on the doxorubicin accumulation in MCF7 cells**. **A: **Cells and spheroids were incubated with 3 μMol/L doxorubicin for 18 h, in the absence (*white columns*) or in the presence (*hatched columns*) of 5 μmol/L YC-1 for 24 h (6 h alone and 18 h together with doxorubicin). Drug accumulation was then measured in MCF7 cell monolayers under normoxic conditions (Monolayer Normoxia), MCF7 3-D spheroids (Spheroids), MCF7 cell monolayers under hypoxic conditions (Monolayer Hypoxia), and MCF7 cells transfected with MDR-1 gene (Monolayer Normoxia Pgp+). ***, P < 0.0001 versus Monolayer Normoxia without YC-1; *, P < 0.05 versus Monolayer Normoxia without YC-1; °°°, P < 0.0001 versus Spheroids or Monolayer Hypoxia without YC-1. **B**: Cells and spheroids were transfected with 400 nM/L of HIF-1α (hatched columns) or transfected with vehicle alone (white columns), and incubated with 3 μMol/L doxorubicin for 18 h. Drug accumulation was then measured in MCF7 cell monolayers under normoxic conditions (Monolayer Normoxia), MCF7 3-D spheroids (Spheroids), MCF7 cell monolayers under hypoxic conditions (Monolayer Hypoxia), and MCF7 cells transfected with MDR-1 gene (Monolayer Normoxia Pgp+). ***, P < 0.0001 versus Monolayer Normoxia transfected with vehicle alone; *; °°°, P < 0.0001 versus Spheroids or Monolayer Hypoxia transfected with vehicle alone.

**Figure 7:**
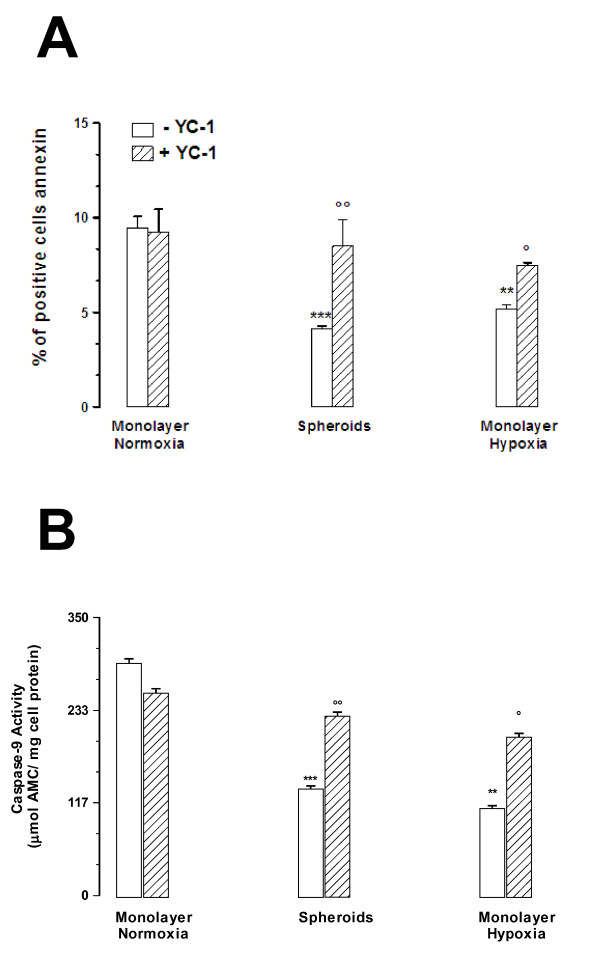
**A: Evaluation of early apoptosis, measured as percentage of cells positive for annexin V by FACS analysis**. Cells and spheroids were incubated with 3 μMol/L doxorubicin for 18 h, in the absence (white columns) or in the presence (hatched columns) of 5 μMol/L YC-1 for 24 h (6 h alone and 18 h together with doxorubicin). Apoptosis was then measured in MCF7 cell monolayers under normoxic conditions (Monolayer Normoxia), MCF7 3-D spheroids (Spheroids), and MCF7 cell monolayers under hypoxic conditions (Monolayer Hypoxia). ***, P < 0.0001 versus Monolayer Normoxia without YC-1; **, P < 0.01 versus Monolayer Normoxia without YC-1; °°, P < 0.001 versus Spheroids without YC-1; °, P < 0.05 versus Monolayer Hypoxia without YC-1. The figures are representative of three similar experiments, performed in duplicate. **B: **Evaluation of apoptosis by detection of caspase activity by fluorogenic assays measured as amount of cleaved fluorogenic substrates from cellular lysates by spectrophotometric analysis. Cleavage of the synthetic fluorogenic substrate Ac-LEDH-AMC by caspase-9. Cells and spheroids were incubated with 3 μMol/L doxorubicin for 18 h, in the absence (*white columns*) or in the presence (*hatched columns*) of 5 μMol/L YC-1 for 24 h (6 h alone and 18 h together with doxorubicin). Apoptosis was then measured in MCF7 cell monolayers under normoxic conditions (Monolayer Normoxia), MCF7 3-D spheroids (Spheroids), and MCF7 cell monolayers under hypoxic conditions (Monolayer Hypoxia). ***, P < 0.0001 versus Monolayer Normoxia without YC-1; **, P < 0.0001 versus Monolayer Normoxia without YC-1; °°, P < 0.001 versus Spheroids without YC-1; °, P < 0.001 versus Monolayer Hypoxia without YC-1. The figures are representative of three similar experiments, performed in duplicate.

After an 18 h-incubation with 3 μMol/L doxorubicin, MCF7 3-D spheroids as well as MCF7 hypoxic cells and MCF7 Pgp + cells accumulated significantly less of the drug than MCF7 control cells (Figure 6). The inhibition of HIF-1α either by incubating cells with YC-1 (5 μMol/L for 24 h) (Figure 6a) or by transfecting cells with specific siRNA for HIF-1α (Figure 6b) increased the doxorubicin accumulation in both MCF7 3-D spheroids and MCF7 hypoxic cells to a level comparable to the one of MCF7 control cells, while it had no effect in MCF7 control cells and, as expected, in MCF7 Pgp + cells. To evaluate the contribution of Pgp in the drug resistance of MCF7 3-D spheroids, we measured drug accumulation in the presence (+) or absence (-) of the Pgp inhibitor verapamil [30]. Verapamil (V, 100 μMol/L) increased intracellular doxorubicin content in MCF7 3-D spheroids (-V: 1840.66 ± 70.58; +V: 4247.30 ± 201.62 ng doxorubicin/mg protein) when compared to MCF7 control cells (-V: 4125.76 ± 89.75; +V: 4088.24 ± 86.96 ng doxorubicin/mg protein, P < 0.0001) (Additional file 1: Figure S1). We also measured the cytotoxic effects of the drug through the detection of cell apoptosis by annexin V-FITC staining. The percentage of annexin V-positive cells exposed to doxorubicin (3 μMol/L, 18 h) was significantly higher in MCF7 control cells than in MCF7 3-D spheroids and in MCF7 hypoxic cells, suggesting in these latter a protection against early apoptosis (Figure 7a). In the presence of YC-1 (5 μMol/L for 24 h), the percentage of annexin V-positive cells increased significantly in MCF7 3-D spheroids and in MCF7 hypoxic cells to a level comparable to the one observed in MCF7 control cells (Figure 7a). It has been reported that doxorubicin induces apoptosis by activating caspase-9 in cancer cells [31], we thus evaluated by fluorogenic assays caspase activity in MCF7 cells exposed to doxorubicin (3 μMol/L, 18 h). We demonstrated that caspase-9 activity was significantly higher in MCF7 control cells than in MCF7 3-D spheroids and in MCF7 hypoxic cells (Figure 7b and 7c). In the presence of YC-1 (5 μMol/L for 24 h), caspase-9 activity increased significantly in MCF7 3-D spheroids and in MCF7 hypoxic cells to a level comparable to the one observed in MCF7 control cells (Figure 7b and 7c). Taken together, these results suggest that MCF7 3-D spheroid formation induced a resistance to doxorubicin, at least in part, via surface Pgp expression.

### Immunophenotypical similarities between of MCF7 3-D spheroids and micropapillary carcinoma

**Figure 8:**
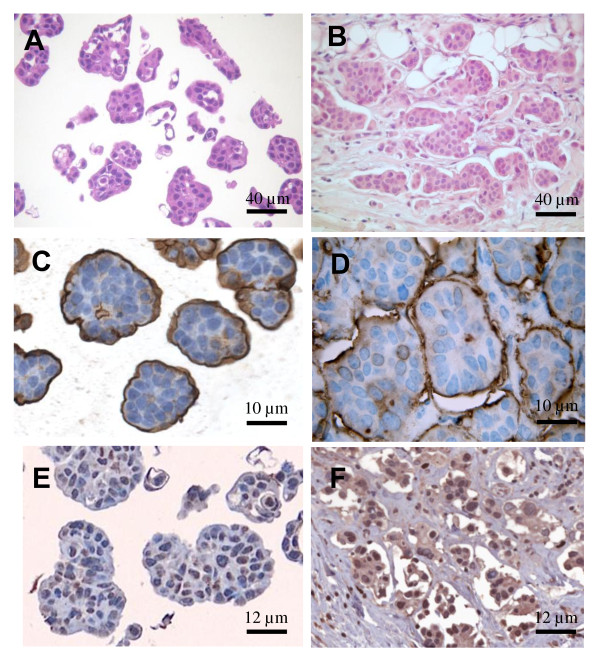
**A and B: Hematoxylin and Eosin staining of formalin fixed paraffin embedded MCF7 3-D spheroids **.  (A, bar 40 microns) The spheroids had almost the same morphology and diameter of the micropapillae of the carcinoma in breast biopsy from a patient with IMPC (B, bar 40 microns). C and D: Immunohistochemical staining for MUC1 in formalin fixed paraffin embedded MCF7 3-D spheroids (C, bar 10 microns) and biopsy from a patient with IMPC of the breast (D, bar 10 microns). E and F: Immunohistochemical staining for HIF1-α in formalin fixed paraffin embedded MCF7 3-D spheroids (E, bar 12 microns) and biopsy from a patient with IMPC of the breast (F, bar 12 microns). The figures are representative of three similar experiments.

**Figure 9:**
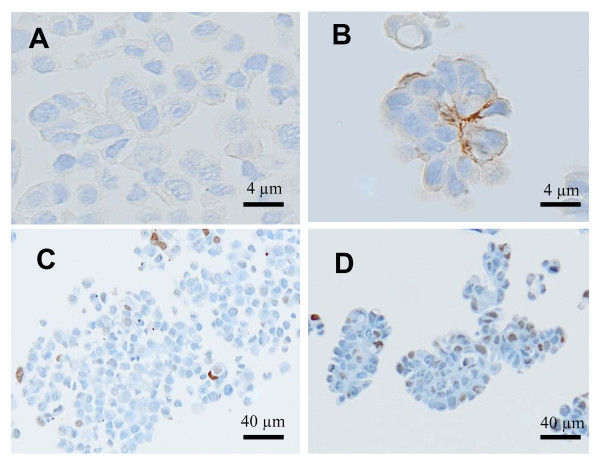
**A and B: Immunohistochemical staining for CD44 in formalin fixed paraffin embedded MCF7 cell monolayers under normoxic conditions.  **. (MCF7 control cells) (A, bar 4 microns) and in MCF7 3D-spheroids (B, bar 4 microns). Spheroids formation induced de novo acquisition of CD44 that was visible on the cell membrane. C and D: Immunohistochemical for p53. Only few nuclei of MCF-7 control cells were positive (C, bar 40 microns), while a higher number of positive nuclei were present in MCF7 3D-spheroids (D, bar 40 microns).

MUC1, a membrane glycoprotein of the apical cell borders, is aberrantly over-expressed by most human breast carcinomas [32]. In IMPC at difference from other breast cancers, MUC1 is expressed on the basal surface of the cell clusters [33,34]. To evaluate whether to the morphological similarity observed between elastase-induced MCF7 3-D spheroids (Figure 8a) and IMPC of the breast (Figure 8b) corresponded also an immunophenotypical similarity, we assessed MUC1 expression in MCF7 3-D spheroids. We demonstrated that in MCF7 3-D spheroids MUC1 was expressed along the outer cell surface of the clusters (Figure 8c) similarly to IMPC (Figure 8d). Both the MCF7 3-D spheroids and the IMPC showed nuclear expression of HIF-1α (Figure 8e and 8f). In addition, we showed that markers of biological aggressiveness known to be expressed in IMPC were also present in MCF7 3-D spheroids: CD44 was expressed in MCF7 3-D spheroids (while absent in MCF7 control cells) and p53 expression was increased compared to the one of MCF-7 control cells (Figure 9).

## Discussion

Neoadjuvant chemotherapy (NAC) is the standard care for locally advanced breast cancer and is used increasingly for large breast cancer with the intention of tumor down-staging so that breast-conserving surgery could be considered as a surgical option [35-37]. IMPC of the breast is a distinct and aggressive variant of breast cancer that shows marked lymphotropism, extensive axillary lymph node involvement at diagnosis, and frequent local recurrence and distant metastases at follow up. In addition, Alvarado-Cabrero et al. have recently reported that none of the IMPC of a case series of locally advanced breast cancers treated by NAC did respond to treatment [7]. To our knowledge, no data exist on the molecular basis of the lack of response to chemotherapy in IMPC of the breast.

In the present study, using a 3-D model that mimics the small papillae typical of the IMPC growth pattern, we demonstrated that HIF-1 activation may be related to the drug resistance described in this tumor. We decided to use the ER-positive MCF7 cancer cells because the IMPC are, in most cases, ER-positive breast cancers.

We first observed that HIF-1α is translocated into the nucleus and is able to bind a HRE both when MCF7 cells are cultured as 3-D spheroids or as monolayers under hypoxic conditions. In contrast, MCF7 cell monolayers under normoxic conditions exhibit a minimal activation of HIF-1. We also showed that expression of *PDK1, PGK1*, and *VEGF*, well-known HIF-1 target genes, was increased in MCF7 3-D spheroids and MCF7 hypoxic cells compared to MCF7 control cells. This observation suggests that in vitro MCF7 3-D spheroid formation may mimic hypoxic conditions and that the activation of HIF-1 in MCF7 3-D spheroids may be an adaptation to hypoxia. This result is in agreement with previous reports showing that cells cultured as 3D-spheroids are associated with a microenvironment characterized by gradients in O_2_, pH, and nutrients, which simulates the architecture of tumors better than the 2-D or monolayer cell culture model [27,28,38].

It has been postulated that hypoxia in solid tumors dramatically decreases the chemosensitivity of tumor cells and that experimental hypoxia promotes drug resistance to anticancer agents in a variety of cell lines [12]. In particular, Comerford et al. revealed that hypoxia induces *MDR-1 *gene expression and concomitant functional expression of its product Pgp in cells grown as monolayers under hypoxic conditions and in cells grown as multicellular spheroids. Moreover, they identified a functional HIF-1 binding site within the *MDR-1 *gene promoter [16]. Later on other groups confirmed that the activation of HIF-1 in tumor cells is associated with an increase in Pgp expression [17-20]. These findings are consistent with the present results reporting that binding of HIF-1α to *MDR-1 *gene promoter is higher in MCF7 3-D spheroids and in MCF7 hypoxic cells than in MCF7 control cells. In addition, we observed an increased expression of Pgp on the surface of cells obtained from 3-D spheroids and from monolayers under hypoxic conditions. This increase is abolished both by the incubation of cells with YC-1, which induces a decrease in HIF-1α accumulation by down-regulating HIF-1α mRNA translation [29], and by the transfection of cells with specific siRNA for HIF-1α. Our results suggest that in both MCF7 3-D spheroids and MCF7 hypoxic cells HIF-1 is activated and is recruited to participate to the transcriptional activity of *MDR-1 *gene, and that the increase of Pgp surface-expression is associated with this activation. It is noteworthy that Pgp is a predominant membrane transporter associated with chemotherapy resistance [39]. Here we demonstrated that MCF7 3-D spheroids and MCF7 hypoxic cells accumulate less doxorubicin and are less sensitive to its cytotoxic effects than MCF7 cells cultured as a monolayer under normoxic conditions. The inhibition of HIF-1α, either by incubating cells with YC-1 or by transfecting cells with specific siRNA for HIF-1α, increases the doxorubicin accumulation in both MCF7 3-D spheroids and MCF7 hypoxic cells to a level comparable to the one of MCF7 control cells, confirming that HIF-1 activation induces doxorubicin resistance. It has been previously demonstrated that doxorubicin induces apoptotic cell death in cancer cell lines [31], even though doxorubicin has been also reported to induce senescence in tumour cells [40]. In the present study, the percentage of annexin V-positive cells and the caspase-9 activity of MCF7 control cells exposed to doxorubicin are low. However, we did not observe in MCF7 control cells enlarged and flattened cells, the predominant morphologic change associated with the onset of drug-induced senescence [41], and we observed significant differences in percentage of apoptotic cells and caspase-9 activity in the following couples: 1) MCF7 control cells and MCF7 3-D spheroids in the absence of YC-1; 2) MCF7 3-D spheroids in the absence of YC-1 and MCF7 3-D spheroids in the presence of YC-1. Moreover, we restricted our interest to doxorubicin because this drug is widely used to treat ER + breast cancer in clinical setting.

Recently, Hung et al. demonstrated that YC-1 is able to modulate the transport function of Pgp and thus to chemosensitize hypoxic tumor cells via the nitric oxide (NO)- cyclic GMP (cGMP)- cGMP-dependent protein kinase (PKG)- extracellular signal-regulated kinase signaling pathway through noncompetitive inhibition [42]. Previous studies have shown that various NO mimetics, including 8-bromo-cGMP, attenuate hypoxia-induced resistance to several chemotherapeutic agents including doxorubicin, suggesting that part of the mechanism of hypoxia-induced drug resistance in cancer cells is the suppression of endogenous NO production [43,44]. Moreover, Frederiksen et al. demonstrated that chemosensitization of hypoxic tumor cells by NO mimetics requires activation of soluble guanylyl cyclase, generation of cGMP, and activation of PKG [45]. Our research group has also reported that a doxorubicin-resistant (HT29-dx) cell population obtained from epithelial colon cell line HT29 accumulates less intracellular doxorubicin, is less sensitive to the cytotoxic effects of the drug, overexpresses Pgp, and exhibits a lower NO production. The resistance to doxorubicin is reversed by inducers of NO synthesis [46]. In the present study, even if we cannot exclude that YC-1 activates the NO-cGMP-PKG signaling pathway, the inhibition of HIF-1α by transfecting cells with specific siRNA for HIF-1α seems to confirm the previously reported inhibitory action of YC-1 on HIF-1α mRNA translation [29].

We demonstrated that the presence of verapamil restores the intracellular doxorubicin content observed in MCF7 3-D spheroids to a level comparable to the one of MCF7 control cells, suggesting that MCF7 3-D spheroid formation induces a resistance to doxorubicin, at least in part, via Pgp. However, verapamil, a commonly used first-generation Pgp inhibitor, is also able to inhibit other ABC transporters such as MDR3 and some multidrug resistance-associated proteins (MRPs) [47]. Verapamil is nevertheless not able to inhibit MRP1 and the breast cancer resistance protein, two other ABC transporters associated with a MDR phenotype in cancer cells [48] and expressed in MCF7 cells (data not shown), suggesting that Pgp may have an important role in the MDR phenotype in MCF7 3-D spheroids.

Finally to confirm that our 3D-culture model produces cell clusters truly similar to IMPC, we evaluated whether the inside-out growth pattern of IMPC was reproduced in vitro [49]. The peculiar architecture of IMPC of the breast has been attributed to a rotation of cell polarization so that the surface of the tumor cells that faces the stroma acquires apical secretory properties and detaches from the stroma. This was shown first by electron microscopy [50] and then confirmed by immunohistochemical staining for MUC1 [33]. MUC1 is a glycoprotein normally located in the apical cell surface of normal glandular epithelium. In conventional carcinomas MUC1 is largely apical, with intracytoplasmic or intercellular distribution [51]. In IMPC, MUC1 has a highly aberrant expression being localized predominantly in the stroma-facing surface of the cells [33,34]. In this study, we obtained in vitro 3-D spheroids by treating MCF7 cells with human neutrophil elastase, as described previously [21]. We demonstrated that the morphological similarity observed between MCF7 3-D spheroids and IMPC of the breast corresponds to a similar distribution of MUC1. Indeed, in MCF7 3-D spheroids MUC1 is expressed along the outer cell surface of the clusters. The mechanism of neutrophil elastase-induced formation of MCF7 3-D spheroids with inverted orientation of epithelial polarity is yet unknown. Moreover, we showed that MCF7 3-D spheroids express markers of biological aggressiveness such as CD44 and p53 similarly to IMPC.

We showed that in breast biopsy from patients with IMPC HIF-1α is expressed in the nucleus of the cells similarly to MCF7 3-D spheroids. This result is in agreement with previous reports showing that ER + breast cancers express strong levels of HIF-1α [52] and its expression is correlated with diagnostic and prognostic indicators for early relapse and metastatic disease [53].

## Conclusions

The present study demonstrates that the MCF7 breast cancer cells cultured as 3-D spheroids are resistant to doxorubicin and that this resistance is associated with an increased Pgp expression at the plasma membrane via activation of HIF-1. The observations made with cultured MCF7 3-D spheroids must be interpreted with caution. Nevertheless, on one hand, MCF7 cells are ER + breast cancer cells, are able to form compact multicellular 3D-spheroids when plated in medium containing human neutrophil elastase, express MUC1 along the outer cell surface of the clusters, and show increased CD44 and p53 levels. On the other hand, IMPC of the breast shows nuclear expression of HIF-1α. So far, the results of the present study suggest that such spheroids could be used as a potential in vitro model to investigate the role of drug resistance in the aggressive behavior of tumor cell clusters encountered in IMPC of the breast.

## Abbreviations

ABC transporter: ATP-binding cassette transporter; AMC: 7-amino-4-methyl coumarin; BSA: Bovine serum albumin; cGMP: Cyclic guanosine monophosphate; ChIP: Chromatin Immunoprecipitation Assay; DTT: Dithiothreitol; ELISA: Enzyme-Linked Immunosorbent Assay; EMSA: Electrophoretic mobility shift assay; ER: Estrogen receptor; FACS: f luorescence-activated cell sorting; FBS: Fetal bovine serum; FITC: Fluorescein isothiocyanate; HIF-1: Hypoxia-inducible factor-1; HRE: Hypoxia response element; IMPC: Invasive micropapillary carcinoma; mAb: Monoclonal antibody; MDR: Multidrug resistance; MRP: Multidrug resistance-associated protein; MUC1: Mucin 1; NAC: Neoadjuvant chemotherapy; NO: Nitric oxide; PBS: Phosphate-buffered saline solution; PVDF: Polyvinylidene fluoride; Pgp: P-glycoprotein; PKG: cGMP-dependent protein kinase; PMSF: Phenylmethylsulfonyl fluoride; RT-PCR: Reverse transcriptase-polymerase chain reaction; SDS-PAGE: Sodium dodecylsulphate-polyacrylamide gel electrophoresis; siRNA: Small interference RNA; TBS: Tris-buffered saline solution; TCA: Trichloroacetic acid; VEGF: Vascular endothelial growth factor; YC: 3-(5'-hydroxymethyl-2'-furyl)-1-benzylindazole.

## Competing interests

The authors declare that they have no competing interests.

## Authors' contributions

SD performed part of the experiments, participated in the coordination of the study, and wrote the final manuscript. DCB performed the main part of the experiments. MP participated in the experiments and in the coordination of the study. SD, DCB, MP, and DG analyzed the data. LA, EA, and CR participated in the experiments. DG reviewed the entire manuscript. AB and AS conceived and designed the study, analyzed the data and helped to draft the manuscript. AS was responsible for histological examination. All authors read and approved the manuscript.

## Pre-publication history

The pre-publication history for this paper can be accessed here:

http://www.biomedcentral.com/1471-2407/12/4/prepub

## Supplementary Material

Additional file 1 **Figure S1 Contribution of Pgp to the doxorubicin resistance in MCF7 cells**. Cells and spheroids were incubated with 3 μMol/L doxorubicin for 18 h, in the absence (white columns) or presence (hatched columns) of 100 μMol/L verapamil (6 h alone and 18 h together with doxorubicin). Drug accumulation was then measured in MCF7 cell monolayers under normoxic conditions (Monolayer Normoxia) and MCF7 3-D spheroids (Spheroids). ***, P < 0.0001 versus Monolayer Normoxia without verapamil; °°°, P < 0.0001 versus Spheroids without verapamil. Click here for file
