# Supplementary figures and images for: HIF-1 activation induces doxorubicin resistance in MCF7 3-D spheroids via P-glycoprotein expression: a potential model of the chemo-resistance of invasive micropapillary carcinoma of the breast
Source: BMC Cancer. 2012 Jan 4;12:4. doi: 10.1186/1471-2407-12-4 (PMC3262753; doi:10.1186/1471-2407-12-4)

## Slide 1
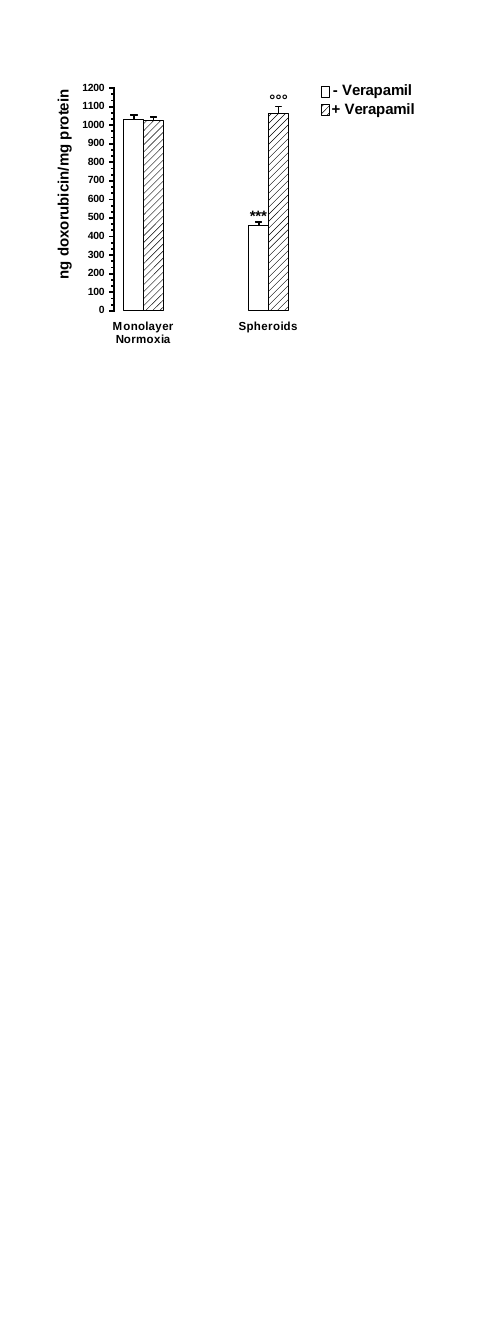

Supplement: Additional file 1 — Figure S1 Contribution of Pgp to the doxorubicin resistance in MCF7 cells. Cells and spheroids were incubated with 3 μMol/L doxorubicin for 18 h, in the absence (white columns) or presence (hatched columns) of 100 μMol/L verapamil (6 h alone and 18 h together with doxorubicin). Drug accumulation was then measured in MCF7 cell monolayers under normoxic conditions (Monolayer Normoxia) and MCF7 3-D spheroids (Spheroids). ***, P < 0.0001 versus Monolayer Normoxia without verapamil; °°°, P < 0.0001 versus Spheroids without verapamil. [file 1471-2407-12-4-S1.PPT]
